# Ketamine administered pregnant rats impair learning and memory in offspring *via* the CREB pathway

**DOI:** 10.18632/oncotarget.15405

**Published:** 2017-02-16

**Authors:** Xinran Li, Cen Guo, Yanan Li, Lina Li, Yuxin Wang, Yiming Zhang, Yue Li, Yu Chen, Wenhan Liu, Li Gao

**Affiliations:** ^1^ College of Veterinary Medicine, Northeast Agricultural University, Harbin, China

**Keywords:** CREB pathway, ketamine, learning and memory, pregnant rats, rat offspring, Neuroscience

## Abstract

Ketamine has been reported to impair the capacity for learning and memory. This study examined whether these capacities were also altered in the offspring and investigated the role of the CREB signaling pathway in pregnant rats, subjected to ketamine-induced anesthesia. On the 14^th^ day of gestation (P14), female rats were anesthetized for 3 h via intravenous ketamine injection (200 mg/Kg). Morris water maze task, contextual and cued fear conditioning, and olfactory tasks were executed between the 25^th^ to 30^th^ day after birth (B25-30) on rat pups, and rats were sacrificed on B30. Nerve density and dendritic spine density were examined via Nissl’s and Golgi staining. Simultaneously, the contents of Ca^2+^/Calmodulin-Dependent Protein Kinase II (CaMKII), p-CaMKII, CaMKIV, p-CaMKIV, Extracellular Regulated Protein Kinases (ERK), p-ERK, Protein Kinase A (PKA), p-PKA, cAMP-Response Element Binding Protein (CREB), p-CREB, and Brain Derived Neurotrophic Factor (BDNF) were detected in the hippocampus. We pretreated PC12 cells with both PKA inhibitor (H89) and ERK inhibitor (SCH772984), thus detecting levels of ERK, p-ERK, PKA, p-PKA, p-CREB, and BDNF. The results revealed that ketamine impaired the learning ability and spatial as well as conditioned memory in the offspring, and significantly decreased the protein levels of ERK, p-ERK, PKA, p-PKA, p-CREB, and BDNF. We found that ERK and PKA (but not CaMKII or CaMKIV) have the ability to regulate the CREB-BDNF pathway during ketamine-induced anesthesia in pregnant rats. Furthermore, ERK and PKA are mutually compensatory for the regulation of the CREB-BDNF pathway.

## INTRODUCTION

Ketamine abuse causes more severe problems during pregnancy [1]. In addition, between 0.75% and 2% of pregnant women require surgery either related to the pregnancy or to unrelated medical problems [2]. Furthermore, with the popularity of minimally invasive surgery, surgery during pregnancy has become increasingly widespread [3]. Therefore, the risks of anesthetic administration (such as ketamine) for the fetus have become more important. Unfortunately, only few reports investigated the effects of generally utilized anesthesia on neurodevelopmental consequences for a fetus prior to birth [4–6].

It is well known that the hippocampus plays a central role for learning and memory processes [7], and consequently, this is a known target for drug regulation. Ketamine is a high affinity uncompetitive antagonist of voltage dependent N-Methyl-D-aspartic Acid Receptor (NMDAR) and has been used for decades as a dissociative anesthetic that also appears to be a useful tool in psychiatric research [8]. Ketamine can attenuate learning and memory impairment, particularly for short-term memory [9]. The cAMP-Response Element Binding Protein (CREB) has been reported to be involved in the learning and memory deficits, caused by ketamine [10].

Ketamine can enter the fetus through the placental barrier, where it may exert a stronger impact on the fetus since the fetal brain is still in a stage of development. Even normal use of ketamine may affect the fetus. Therefore, this study examined whether the abilities of learning and memory were altered in the offspring as well as the ketamine-induced effect on CREB signaling pathways in ketamine-induced pregnant rats on gestational day 14.

## RESULTS

### Nissl's staining

We selected three 10^4^ μm^2^ areas to conduct a neuron count in both the CA1 (Figure 2c-2d) and CA3 (Figure 2e-2f) regions of the hippocampus. As shown in Figure 2a-2g, the cell density of K group decreased by 20.5% compared to C group (*p* < 0.05).

**Figure 1:**
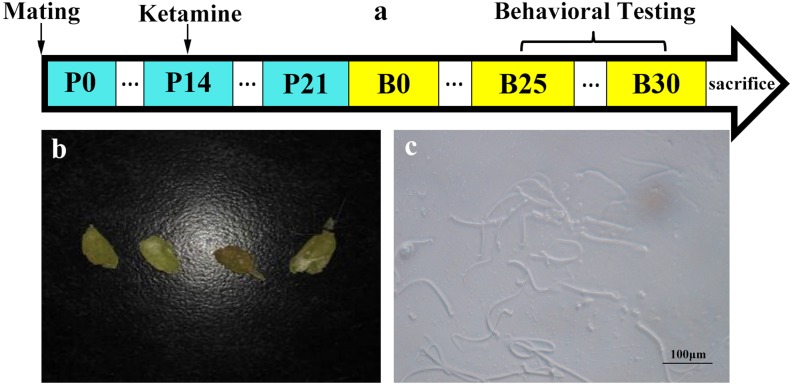
Mating and drug administration The Vaginal suppository (**b**) and sperm (**c**) were observed and female rats were defined as pregnant at day 0 (P0). Female rats were anesthetized via intravenous ketamine injection on P14. The first day after birth was recorded as B0. During B25-B30, behavioral testing was utilized to test the learning and memory capacities (**a**).

**Figure 2:**
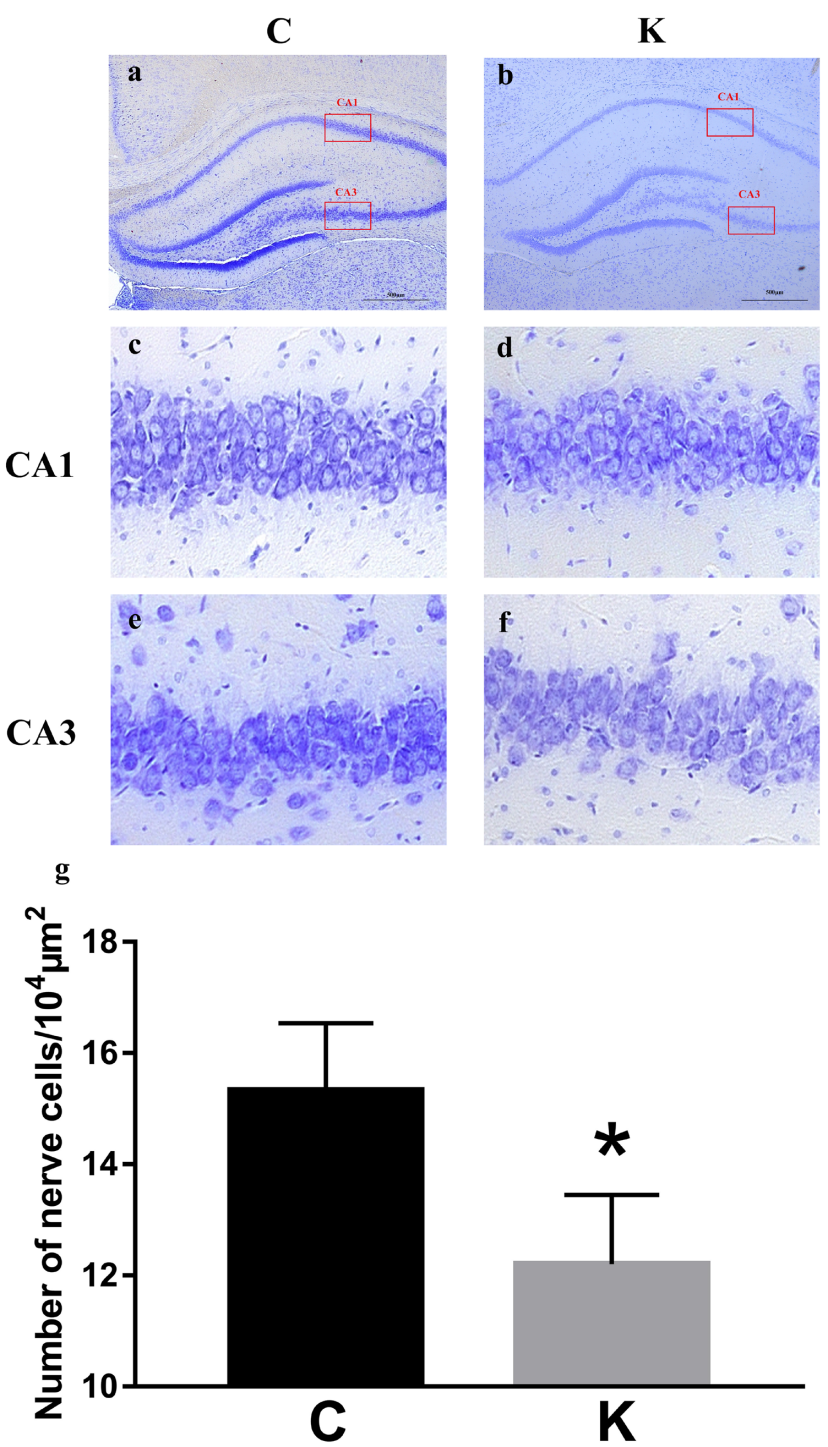
Nissl's staining was utilized to observe neuronal cells **a**. and **b**. Areas of 10^4^ μm^2^ were selected and neuron numbers were counted in the CA1 **c**. and **d**. and CA3 **e**. and **f**. regions of the hippocampus. **g**. Cells within K group decreased compared to C group (*p* < 0.05).

### Golgi staining

**Figure 3:**
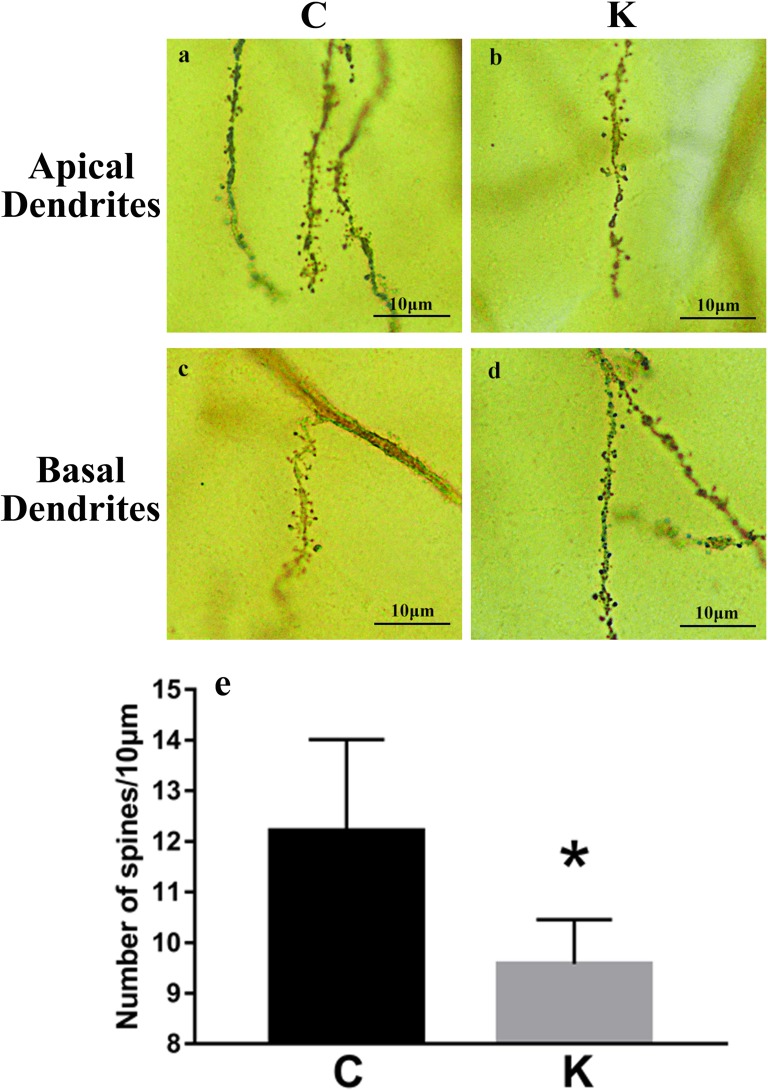
Golgi staining revealed hippocampal dendritic spine density 10 μm of apical **a**. and **b**. and basal **c**. and **d**. dendrites were randomly selected from each pyramidal neuron for inspection (via 200 × oil immersion lens) to quantify spinal density. **e**. Spinal density decreased by 21.6% in K group compared to C group (*p* < 0.05).

Fully impregnated CA1 pyramidal cells can be detected via Golgi staining, and the spines of the apical dendrites can be analyzed under a light microscope using a 200 × oil immersion objective. We randomly selected 10 μm apical (Figure 3a-3b) and basal (Figure 3c-3d) from the same neurological level to count the number of dendritic spines. Only the density of apical and basal dendrites was determined in our study, as several different types of spines were not always clearly visible (e.g., thin, mushroom, or branched dendrites). Spine density decreased by 21.6% in the K group compared to the C group (*P* < 0.05, Figure 3g).

### Morris water maze test

**Figure 4:**
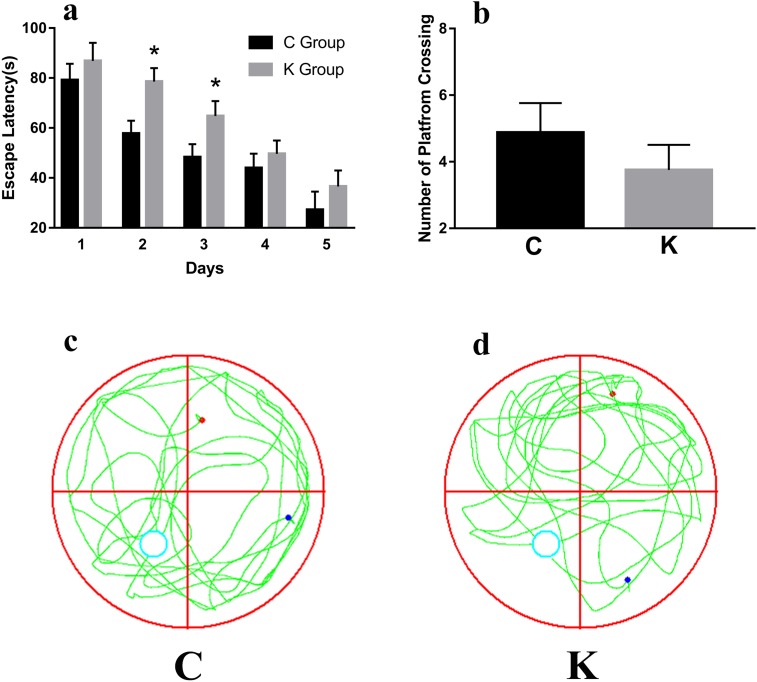
To test hippocampus-dependent spatial cognition, rats were trained in the standard morris water maze Rat pups showed significant main effects in their escape latency during spatial training during testing days 2-3 **a**. During the 60 s probe trial, we recorded and analyzed the swimming path tracks **c**. and **d**.; however, no significant differences were detected in the spatial probe test **b**.

Morris water maze test data revealed that rat pups had suffered from a significant main effect on escape latency during spatial training on testing days 2-3 (Figure 4a). In contrast, the escape latency did not reveal any significant differences between the C group and the K group during place navigation trials on day 4-5 (*p* = 0.223) and spatial probe tests (*p* = 0.062, Figure 4b). It is worth noting that no significant differences in animals’ swimming speeds were detected (C group: 25.00 ± 1.96 cm/s and K group: 26.00 ± 1.33 cm/s, according to a one-way ANOVA: F = 0.191, *p* = 0.827) between the C and K group (Figure 4c-4d).

### Contextual and cued fear conditioning

**Figure 5:**
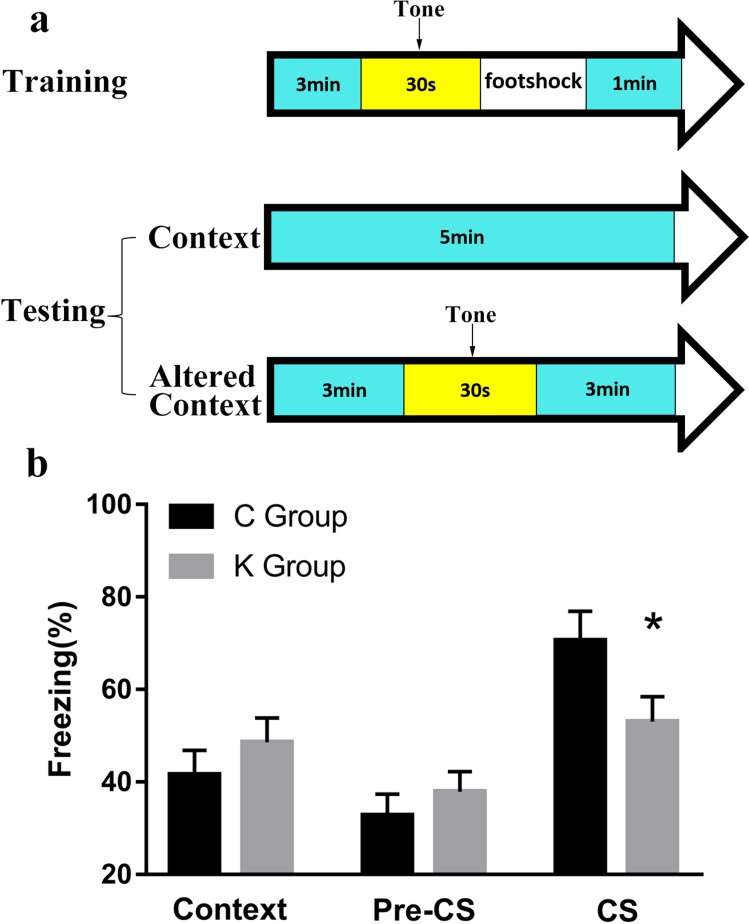
Contextual and cued fear conditioning is a fear conditioning task that measures the ability of a rat to learn and remember an association between an aversive experience and environmental cues **a**. Experimental process of contextual and cued fear conditioning. **b**. A significant difference was found in CS between K and C group.

Contextual and cued fear conditioning is a standard fear conditioning task that measures the ability of rat pups to learn and remember an association between an averse experience and environmental cues. In contextual and cued fear conditioning, a significant difference was found in CS (*p* < 0.05), while no significant difference was found between K and C group in other tests (Figure 5b).

### Olfactory tasks

**Figure 6:**
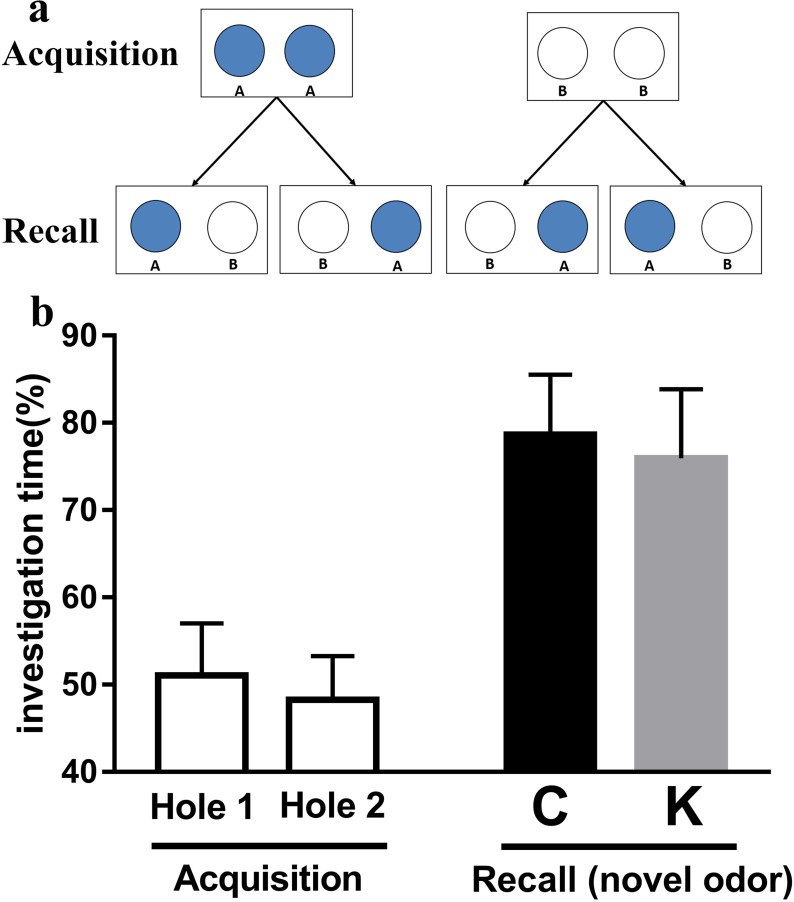
Olfactory discrimination tasks are excellent measures of learning and memory in rats The acquisition test (one session) consisted of presentation of one odor (limonene or carvone), presented in both holes. The recall test consisted of a 3 min session in which one hole was odorized with the previously presented odor, while the other hole with a novel odor (a). There was no significant difference in the investigation time of novel odor between K group and C group (b).

As shown in Figure 6b, during the acquisition stage, no significant differences were found in the investigation time of Hole 1 and Hole 2, indicating that rats had no specific preference for carvone or limonene. During the recall stage, no significant differences were found in the investigation time of novel odor between K group and C group.

### Results of the CCK-8 test

To evaluate the optimum concentration of ketamine for PC12 cells, a CCK8 assay was performed. PC12 cells were exposed to ketamine at different concentrations (0.2 μg/mL, 0.3 μg/mL, 0.4 μg/mL, 0.5 μg/mL, 0.6 μg/mL, 0.7 μg/mL, 0.8 μg/mL, 0.9 μg/mL, 1μg/mL) for 3 h. According to Table 1, the 50% cell viability of ketamine for PC12 cells was 0.6 μg/mL.

**Table 1:** Results of CCK-8 test

Concentration of ketamine (μg/mL)	1	0.9	0.8	0.7	0.6	0.5	0.4	0.3	0.2
Cell viability	17%	34%	39%	43%	49%	53%	64%	75%	76%

### Ketamine exposure affects protein expression in the hippocampus

**Figure 7:**
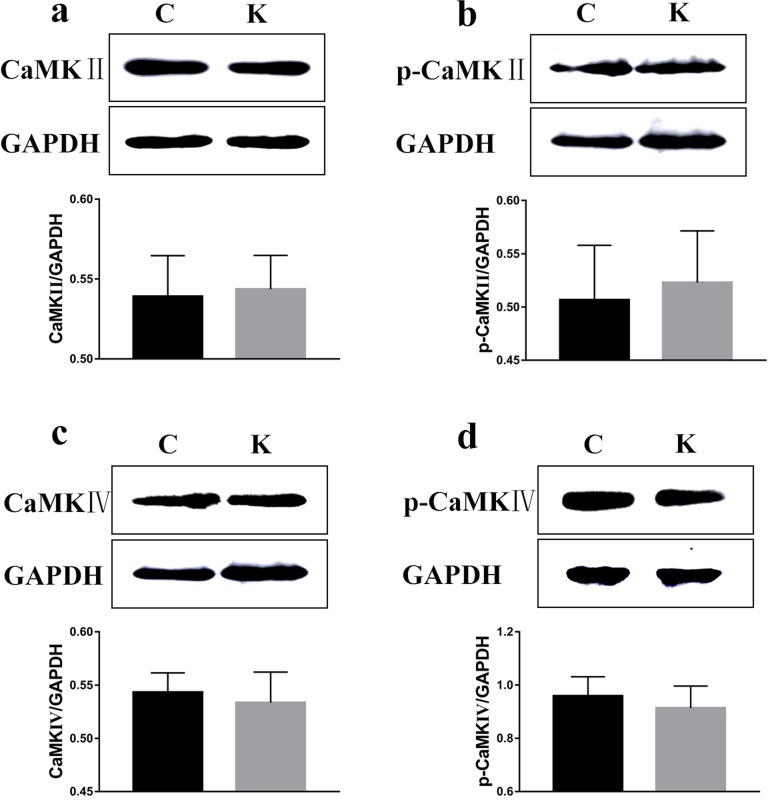
Ketamine exposure affects protein expression in the hippocampus No signifcant difference was found in the protein levels of CaMKII. **a**., p-CaMKII **b**., CaMKIV **c**., and p-CaMKIV **d**.

**Figure 8:**
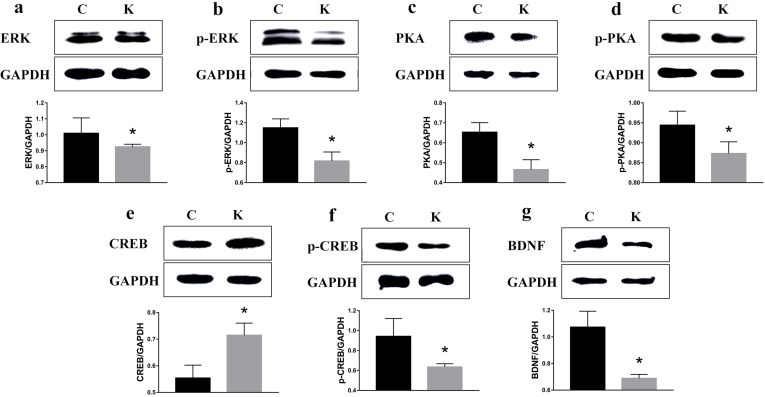
Ketamine exposure affects protein expression in the hippocampus Protein levels of ERK **a**., p-ERK **b**., PKA **c**., p-PKA **d**., p-CREB **f**., and BDNF **g**. signifcantly decreased in the hippocampus (*p* < 0.05) to 91.6%, 71.1%, 74.5%, 92.5%, 67.4%, and 64.2%, of their initial values, while the CREB **e**. protein level signifcantly increased to 129%.

To examine whether ketamine treatment has the ability to alter the protein expression of learning and memory related proteins, we measured protein levels of CaMKII, p-CaMKII, CaMKIV, p-CaMKIV, ERK, p-ERK, PKA, p-PKA, p-CREB, and BDNF in the rat hippocampus. As shown in Figure 7 and in comparison to the values of each corresponding C group, no significant difference was found in the protein levels of CaMKII, p-CaMKII, CaMKIV, and p-CaMKIV. However, the protein levels of ERK, p-ERK, PKA, p-PKA, p-CREB, and BDNF had significantly decreased (*p* < 0.05) to 91.6%, 71.1%, 74.5%, 92.5%, 67.4%, and 64.2% of their original values, respectively, while the CREB protein level significantly increased (*p* < 0.05) to 129% (Figure 8).

### Ketamine exposure affects protein expression in PC12 cells

**Figure 9:**
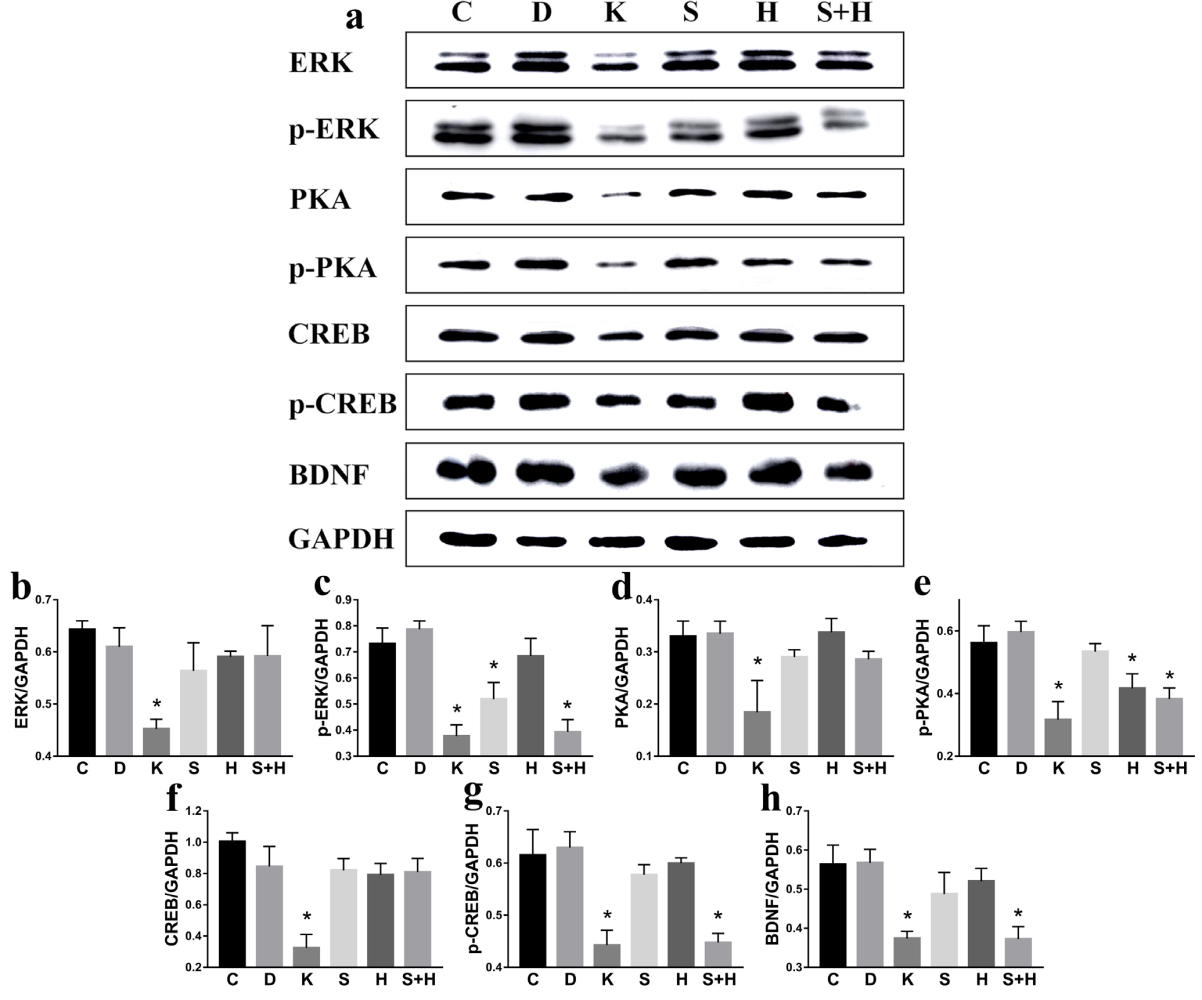
Ketamine exposure affects protein expression in PC12 cells C: Control group; D: D group, DMSO (solvent of inhibitors); K: K group, ketamine; S: S group, SCH772984 (ERK inhibitor); H: H group, H89 (PKA inhibitor); S+H: S+H group (PKA inhibitor + ERK inhibitor). **a**. No significant difference was found between C and D group. Compared to the C group, the protein levels of ERK **b**., p-ERK **c**., PKA **d**., p-PKA **e**., CREB **f**., p-CREB **g**., and BDNF **h**. decreased significantly (*p* < 0.05).

No significant difference was found between C and D group. Compared to C group, the protein levels of ERK, p-ERK, PKA, p-PKA, CREB, p-CREB, and BDNF decreased significantly (*p* < 0.05): p-ERK decreased to 70.9% in S group (*p* < 0.05), p-PKA decreased to 74.4% in H group (*p* < 0.05), and the protein levels of p-ERK, p-PKA, p-CREB, and BDNF decreased to 53.7%, 68.1%, 72.7%, and 66.1% (*p* < 0.05). The protein levels of p-ERK decreased by 24.3% in S+H group compared to S group, while the p-PKA protein levels decreased by 8.5% in S+H group compared to H group (Figure 9).

## DISCUSSION

The morris water maze task, contextual and cued fear conditioning, and olfactory tasks enable the evaluation of learning and memory of spatial, conditioned, and odor cues in rat pups (Figure 4–6). Nerve density and dendritic spine density decreased, which all influenced the nerve conduction efficiency as well as learning ability and memory capacity [6, 11]. However, during the anaesthetization process, anesthetic stress, oxygen saturation, gastrointestinal tract squeezing the uterus, change of placental blood flow, supine position, oppressing blood vessels, and other factors all impact on fetal rats. To exclude these effects from our analysis, we cultured PC12 cells, exploring the effect of ketamine on the CREB pathway.

The prevalence of substance abuse in pregnant women is similar to that of the general population, resulting in an increased fetal exposure rate during the most vulnerable period of neurodevelopment and organogenesis. Many pregnant women are exposed to various types of anesthetics for surgery or diagnostic procedures every year. Furthermore, numerous women will also undergo surgery during pregnancy, unrelated to childbirth. Consensus is that fetal exposure to alcohol is harmful. Prenatal alcohol exposure may induce abnormal brain development as well as decrease the capacity for learning and memory [12, 13]. Similar to alcohol, anticonvulsants, sedatives (such as ketamine), or narcotics can pass through the placental barrier and for ketamine in particular, researches showed its ability to impair the capacity for learning and memory [14, 15]. Evidence links early exposure to anesthesia with cognitive impairment [16]. In addition, ketamine is also one of the most commonly used drugs in pediatric clinical anesthesia and its reported influence on learning and memory has always been of clinical concern. Moreover, ketamine is a frequently abuse drug in the public, which includes pregnant women [17]. Zhang et al. suggests that repeated ketamine exposure induced long-term cognitive impairment via increased NOX2 [18]. Experimental evidence indicates that the NMDAR antagonist ketamine impairs cognition [19]. Prolonged ketamine exposure in neonates at anesthetic doses has been reported to cause long-term impairments of learning and memory [20]. Furthermore, ketamine decreased p-CREB in the hippocampus [21, 22], and decreased levels of BDNF [23]. CREB has been demonstrated to be involved in learning and memory deficits caused by ketamine [10, 22]. These findings raise concern about potential adverse effects of ketamine exposure to fetuses and infants.

In our study, the ratio of P-CREB/total CREB was decreased in the rat hippocampus. The function of CREB is dominantly regulated by phosphorylation at Ser133, which results in the activation of gene transcription [24]. The Phosphorylated CREB protein recruits the transcriptional activator CREB-binding protein (CBP), thus stimulating the transcription of CRE-regulated genes that are involved in neurogenesis and neuritogenesis [25]. We therefore hypothesize that P-CREB may be responsible for compensatory increases in CREB protein levels; however, further testing is required to confirm this hypothesis. p-CREB promotes immediate early genes such as the c-fos gene via interaction with the CRE sequence located within promotor regions (TGACGTCA) [26]. Genetic deletion of CREB selectively impaired the hippocampus-dependent spatial memory of mice subjected to the Morris water maze [27], which coincided with our results. Furthermore, CREB phosphorylation is a necessary step in the process leading to the generation of new dendritic spines [27]. In addition, the cAMP–CREB signaling cascade is critical for the generation of new neurons in the rodent hippocampus, also facilitating their subsequent morphological maturation [28].

Several findings have shown that the dysregulation of the CREB-BDNF cascade has been involved in cognitive impairment [29]. The neurons of the hippocampus of aged animals showed a down-regulation of BDNF and p-CREB expression, associated with learning and memory impairment [30, 31], which was also similar with our result. In this study, BDNF and p-CREB revealed the same tendency (Figure 9g-9h). BDNF has also been reported to elicit rapid action potentials, thus influencing neuronal excitability, and it has a demonstrated role in activity-dependent synaptic plasticity events such as long-term potentiation, learning tasks, and memory [32, 33]. BDNF is involved in structural remodeling, neuronal plasticity, and synaptic restructuring [34, 35].

**Figure 10:**
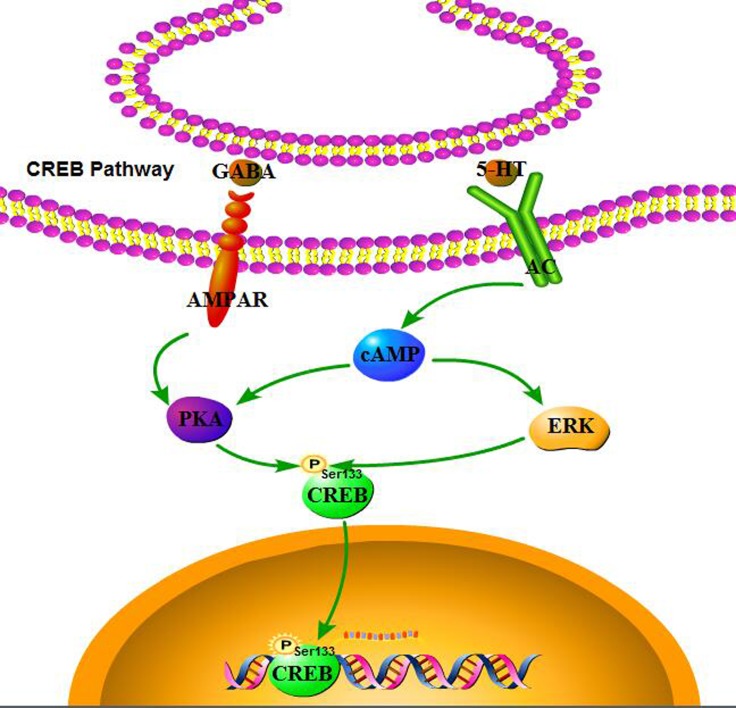
CREB pathway Several signaling pathways, including those involving ERK, and PKA have been associated with the regulation of *de novo* protein synthesis in the context of synaptic plasticity, converging on the phosphorylation of CREB at Ser133 residue.

Several signaling pathways, including those involving CaMKII, CaMKIV, ERK, and PKA, have been associated with the regulation of *de novo* protein synthesis in the context of synaptic plasticity, converging on the phosphorylation of CREB at Ser133 residue (Figure 10). It is generally accepted that ketamine blocks NMDAR, thus mediating the neurotransmission of postsynaptic receptors [36]. NMDAR in turn mediates the release of neurotransmitters (such as acetylcholine, dopamine, GABA, and NE), and regulates the levels of sodium and calcium. The increased association between CaMK II and CREB, followed by phosphorylation of CREB in response to Wnt5a stimulation was suppressed in a minimal hepatic encephalopathy rat model [37]. Ca^2+^ signaling not only plays a critical role in regulating apoptosis and autophagy [38], but also affects CREB phosphorylation. Cohen reported that CREB phosphorylation also proceeds with slow, sigmoid kinetics, that are rate-limited due to the paucity of CaMKIV, protecting against saturation of phospho-CREB as a response to increased firing rates and elevated Ca^2+^ transients [39]. Interestingly, no significance was found in the protein levels of CaMKII, p-CaMKII, CaMKIV, and p-CaMKIV in rat pups (Figure 7). Liu's research demonstrated that low-intensity pulsed ultrasound increased the intracellular concentration of calcium and enhanced protein levels of CaMKII and CaMKIV; however, it did not promote the activation of CREB [40]. CaMKII was markedly decreased following a stress-priming methamphetamine-induced conditioned place preference reinstatement test; however, p-CREB expressions in the medial prefrontal cortex were increased [41]. Guo reported that ERK and CREB phosphorylation was not mediated by CaMK [42]. Therefore, we speculate here that the effect of ketamine on CaMKII and CaMKIV can only be sustained within a window of time following anesthesia. Consequently, it did not have long-term effects on neurodevelopment, but a proving experiment is still required.

To explore whether ERK or PKA influence the phosphorylation of CREB, we cultured PC12 with both a ERK and a PKA inhibitor (SCH772984 and H89). PKA phosphorylates and activates CREB, which then binds to the CRE domain on DNA and in turn activates genes that are involved in the process of learning and memorization; however, ketamine inhibits this process [10]. N-acetylserotonin appears to partly restore the ketamine-induced decrease of ERK and BDNF to control levels [43]. Phosphorylation of CREB at Ser133 can be catalyzed via a number of protein kinases, including cAMP-dependent PKA [44]. The ERK1/2 are members of the mitogen activated protein kinase (MAPK) family and are necessary for cell growth, differentiation, survival, molecular information processing, and stabilization of structural changes in dendritic spines [45, 46]. When treated with SCH772984 or H89 alone, no change in the protein levels of p-CREB and BDNF were observed; however, these decreased when treated with the SCH772984 or H89 (Figure 9g-9h). This explains how ERK and PKA can regulate the phosphorylation of CREB. Furthermore, when ERK or PKA do not participate in this process, ERK and PKA have the ability to replace each other, thus independently regulating the phosphorylation of CREB. Lin identified the involvement of cAMP/PKA and ERK dependent CREB signaling pathways in the luteolin-mediated miR-132 expression and neuritogenesis of PC12 cells [47]. Won demonstrated that DA-9801 exerts its beneficial effects of stimulating neurite outgrowth through the ERK1/2-CREB pathway in PC12 cells [48]. Behavioral analyses of animals with altered ERK signaling have revealed a central involvement of this cascade in learning and memory [49], and it has been reported that ERK activity was decreased in dentate gyrus of aged rats, which did not sustain LTP [50, 51]. Coccomyxa gloeobotrydiformis (CGD) significantly increased ERK and CREB phosphorylation in the hippocampus, suggesting that the learning and memory-enhancing effects of CGD might be associated with the ERK/CREB pathway [52]. Li et al. reported that pretreatment with resveratrol effectively restored synaptic plasticity in chronic cerebral hypoperfusion rats both functional and structural via PKA-CREB activation [28]. The levels of PKA and cAMP were increased in the rat hippocampus following a step-down inhibitory avoidance task [53]. Furthermore, transgenic mice with the inhibitory regulatory subunit of PKA were impaired in their long-term memory abilities due to contextual fear conditioning [54].

In summary, the present study investigated learning as well as spatial and conditioned memory of rat pups, following ketamine anesthesia during pregnancy. Moreover, ERK and PKA, but not CaMKII or CaMKIV, can regulate the CREB-BDNF pathway in this animal model. Furthermore, ERK and PKA in the regulation of CREB-BDNF pathway are mutually compensating.

## MATERIALS AND METHODS

### Animals

Male and female Wistar rats, three months of age, weighing 200 ± 20 g, were purchased from the Animal Experimental Center of the Second Affiliated Hospital of the Harbin Medical University (Harbin, China). Prior to the experiment, rats were quarantined for two weeks at the Northeast Agricultural University (Harbin, China). All experiments were performed in accordance with the guidelines outlined by the Ethical Committee for Animal Experiments (Northeast Agricultural University, Harbin, China).

### Mating and drug administration

Thirty-six Wistar rats were divided into 12 cages (one male and two females per cage) with an iron mesh at the bottom. On the next morning the vaginal suppository was investigated through the iron mesh. When sperm was detected, female rats were annotated as pregnant at day 0 (P0). The female rats were anesthetized via intravenous ketamine injection (200 mg/Kg) for 3 h on P14 [55]. The total volume of ketamine stayed below 2 mL/100 mg. Ketamine-treated offspring were recorded as K group, while individuals within the control group were recorded as C group. The first day after birth was recorded as B0. During B25-B30, Morris water maze task, contextual and cued fear conditioning, and olfactory tasks were used to test learning and memory capacity (n = 120, 5/dam, Figure 1).

### Sample collections

Rat pups were sacrificed at B30 via cervical dislocation, and were recovered to collect brain tissue for Nissl staining (n = 24, 1/dam), Golgi staining (n = 24, 1/dam), and western blotting (n = 72, 3/dam). A subset of their hippocampuses were quickly dispensed on ice, put into a freezing tube, and frozen in liquid nitrogen, while other tissues were preserved in 10% formalin.

### Nissl's staining

Coronal brain sections were cut in a vibratome (Leica VT1200S, Germany) after the brains were postfixed in the same fixative. To ensure matching of hippocampal sections between groups, we used anatomical landmarks provided by the brain atlas. The selected brain sections were stained with 0.5% cresyl violet and we selected three 10^4^ μm^2^ areas for examination with a light microscope (Leica DFC420, Germany) to count neuron numbers in the CA1 and CA3 regions of the hippocampus.

### Golgi staining

Golgi-Cox staining was utilized to obtain hippocampal dendritic spine density via the FD Rapid GolgiStainTM Kit (FD Neuro Technologies Inc), following the manufacturer's instructions. Coronal tissue sections of 150 μm thickness were cut at room temperature, using a vibratome (Leica VT1200S, Germany) and then, they were put on gelatin coated slides. Subsequently, slides were dehydrated with a gradient of 50%, 75%, 95%, to 100% ethanol and cleared in xylene, then the specimens were prepared with slide coverslips and sealed with Permount. The slides were then examined in detail with a light microscope (Leica DFC420, Germany). We analyzed the stained spine, using techniques similar to those described in previous study [56]. Five pyramidal neurons were analyzed that were well-impregnated and clearly distinguishable from others in each hippocampus (20 × objective lens). Five segments of 10 μm of apical and basal dendrites respectively, were randomly selected from each pyramidal neuron for inspection (via 200 × oil immersion lens) to quantify the density of spines. Spinal density of secondary apical and basal dendrites was analyzed at proximal segments emerging at more than 50 μm distance from the soma of the hippocampal CA1 neurons. All of these spines were required to exhibit a clearly distinguishable base or origin and were isolated from neighboring dendrites. Spine density was calculated per 10 μm of dendritic length. The open-source ImageJ 1.48 r Java image-viewing software and Adobe Photoshop CC 2015 were used to calibrate the scale and enlarge the segments of the spines. An investigator blinded to the experimental condition completed all analyses.

### Morris water maze test

### Place navigation trials

To test hippocampal-dependent spatial cognition, rats were trained in the standard morris water maze with a hidden platform [57]. A white escape platform (12 cm diameter) was submerged in a circular pool (160 cm diameter, at a 50 cm depth), filled with warm (23–25°C) opaque water. At B25-29, each rat pup underwent four trial sessions per day (60–70 min inter-trial interval) for five consecutive days. Each trial consisted of releasing the rat into the water, facing the outer edge of the pool at one of the quadrants (in random sequence) and permitting the animal to escape to the platform. They received four trials per day of training in search for the submerged and unmarked platform, with trial durations of 60 s on the platform at the end of trials. All trials were videotaped, and the swimming paths of rats were recorded with the ANY-maze video tracking system (Stoelting Co., IL, USA), which enabled us to measure the time taken (latency) to find the platform (s), as well as other behavioral information obtained during this spatial reference memory test. The animals were dried and placed beneath a heating lamp after completion of each test.

### Spatial probe test

A probe trial was performed 1 d after the last trial at B30 where the platform was removed from the pool to assess memory retention for the location of the platform. During the 60 s test trial, we recorded and analyzed the swimming speed (cm/s), the swimming path tracks, and the number of entries into the platform quadrant zone.

### Contextual and cued fear conditioning

Conditioning training on day one consisted of placing the rat pups in the chamber and exposing the animals to a mild footshock paired with an auditory cue. The rat pup was brought from the home cage to the testing room and placed into the conditioning chamber. It had 3 min to explore the novel environment. The auditory cue (a 90 dB tone) was sounded for approximately 30 sec. A stimulus light within the wall of the chamber may also be illuminated. During the last seconds of the auditory signal, an unconditioned aversive stimulus, a mild footshock in the range of 0.25 to 0.5 mA, was administered through the grid floor for 2 sec. The number of seconds spent freezing in the test chamber on the training day was considered the control measure of unconditioned fear. The rat pup was left in the conditioning chamber for 1 min after the last pairing, during which the association between the aversive stimulus and the properties of the conditioning chamber was further established. The rat pup was then returned to its home cage.

Testing on day 2 began approximately 24 hours after the conditioning session. The rat pup was returned to the same conditioning chamber and scored for bouts of freezing behavior. No footshock was administered on day two. The number of seconds spent freezing in the identical test chamber on day two was considered the measure of contextually conditioned fear, i.e., freezing within identical context. Freezing was defined as a lack of movement other than respiration. Presence or absence of freezing behavior was generally recorded by an investigator, who was blinded to the experimental condition, taking a note every 10 sec for 5 min, for a maximum total score of 30 freezing bouts. The rat pup was then returned to its home cage.

The second phase of testing began an hour later. A further testing chamber with very different properties provided the altered context. Changing the sensory cues as much as possible was essential so that the rat pup perceives the novel context as unrelated to the conditioning chamber. Such as triangle-shaped test chamber with different lighting was used and lemon juice was painted on the walls, while a different investigator wore gloves and a lab coat of different texture than on the training day. Freezing behavior was scored for 3 min. Contextual discrimination of fear conditioning was quantified by comparing the number of freezing bouts in the same contextual environment to the number of freezing bouts in the novel contextual environment.

At the end of the first 3 min, the tone that was presented on training day one (was well as the light stimulus cue if used on day one) was presented in the novel context environment. Freezing behavior was scored for the next 3 min in the presence of the sound (and light) cues. Cued conditioning was calculated via comparison of the number of freezing bouts in the novel context environment in the presence of the cue with the number of freezing bouts in the novel context environment in the absence of the cue (Figure 5a).

### Olfactory task

This task was designed to investigate the olfactory learning and memory abilities [58]. For this experiment, two holes (3 cm diameter and 4.5 cm deep) were used. A polypropylene swab, embedded in a fine plastic mesh and containing 20 μL of diluted odors (1:10) was placed at the bottom of each hole and covered with wood shavings. The acquisition test (one session) consisted of one odor (either limonene or carvone, Sigma-Aldrich) being presented in both holes for a 5 min period. In a preliminary experiment, with simultaneous presentation of the same pair of odors (one odor in each hole) in a one-trial test, rat pups spent the same amount of time exploring either hole, indicating no preference for one of the two odors. The recall test consisted of a 3 min session in which one hole was odorized with the previously presented odor, while the other hole was odorized with a new odor (Figure 6a). The delay between acquisition and recall tests was 60 min. During the recall test, the cumulated exploration time of each hole was converted as the percentage of the total exploration time of both holes. Rat pups were considered to have remembered the familiar odor when they spent less time exploring the hole containing it, in relation to the time spent exploring the hole containing the new odor. Equal exploration times for both holes during the recall test were considered to indicate that rat pup did not remember the familiar odor. Both odors were used alternatively during acquisition or recall and presented randomly in each of the two holes to avoid place preference bias (Figure 6a).

### Cell culture and drug treatment

PC12 cells were obtained from the Northeast Agricultural University, Harbin, China. The cells were cultured in DMEM medium (Gibco), supplemented with 10% (v/v) FBS, penicillin/streptomycin (100 U/mL; 100 μg/mL) at 37°C under an atmosphere of 5% CO_2_ and 95% air. The cells were seeded in 6-well plates with 2-9 × 10^5^ cells/well or 96-well plates with 2-9 × 10^4^ cells/well, and the culture medium was changed daily. Cells were pretreated for 3 h with Protein Kinase A (PKA) inhibitor (H89, 10 μM, H group), Extracellular Regulated Protein Kinases (ERK) inhibitor (SCH772984, 10 μM, S group), PKA inhibitor + ERK inhibitor (S+H group), DMSO (solvent of inhibitors, D group), and ketamine (K group).

### Cell counting kit-8 (CCK-8) assay

Cell viability was detected via the CCK8 assay (Beyotime Institute of Biotechnology, Suzhou, Jiangsu, China). Following the indicated treatments, CCK8 solution (10 μl) was added to each well (96-well plates). Then, the cells were cultured at 37°C for one further hour. The optical density of each well was measured at 450 nm with a Bio-Tek microplate reader (Bio-Tek Instruments, Thermo Fisher Scientific, Winooski, VT).

### WB

150 μg of protein were separated via 10% SDS-polyacrylamide gel electrophoresis and transferred to a nitrocellulose membrane (HybondTM-C Extra, GE Healthcare) via electroblotting. After washing, membranes were blocked with 3% (w/v) BSA (biotopped) for 4 h at room temperature and incubated overnight at 4°C in BSA with antibodies that are specific for Ca^2+^/Calmodulin-Dependent Protein Kinase II (CaMKII), p-CaMKII, CaMKIV, p-CaMKIV, ERK, p-ERK, PKA, CREB, p-CREB (1.5:1000, EnoGene), p-PKA, and Brain Derived Neurotrophic Factor (BDNF, 1:1000, abcam). Membranes were washed thrice with PBS containing 0.1% Tween and then incubated for 1 h at room temperature either with a horseradish peroxidase-conjugated secondary antibody (Goat anti-Rabbit IgG Antibody HRP (ABIN) or a goat anti-Mouse IgG Antibody HRP (Sigma)) in BSA.

### Data analysis

All data were analyzed with GraphPad Prism 7.0 (GraphPad Software Inc., USA) via one-way ANOVA, followed by Turkey's Post Hoc test or unpaired two-tailed Student t-test. Values were considered to be statistically significant for P < 0.05. Data are presented as means ± standard deviation unless otherwise noted.
